# One ion to catch them all: Targeted high-precision Boltzmann thermometry over a wide temperature range with Gd^3+^

**DOI:** 10.1038/s41377-021-00677-5

**Published:** 2021-11-22

**Authors:** Dechao Yu, Huaiyong Li, Dawei Zhang, Qinyuan Zhang, Andries Meijerink, Markus Suta

**Affiliations:** 1 Engineering Research Center of Optical Instrument and System, The Ministry of Education, Shanghai Key Laboratory of Modern Optical Systems, University of Shanghai for Science and Technology, Shanghai, 200093 China; 2 Condensed Matter and Interfaces, Debye Institute for Nanomaterials Science, Department of Chemistry, Utrecht University, Princetonplein 1, 3584 CC Utrecht, The Netherlands; 3 School of Materials Science and Engineering, Liaocheng University, Liaocheng, 252059 China; 4 State Key Laboratory of Luminescent Materials and Devices, and Institute of Optical Communication Materials, South China University of Technology, Guangzhou, 510641 China; 5 Inorganic Photoactive Materials, Institute of Inorganic Chemistry, Heinrich Heine University Düsseldorf, Universitätsstraße 1, 40225 Düsseldorf, Germany

**Keywords:** Optical sensors, Fluorescence spectroscopy, Imaging and sensing

## Abstract

Ratiometric luminescence thermometry with trivalent lanthanide ions and their 4f^*n*^ energy levels is an emerging technique for non-invasive remote temperature sensing with high spatial and temporal resolution. Conventional ratiometric luminescence thermometry often relies on thermal coupling between two closely lying energy levels governed by Boltzmann’s law. Despite its simplicity, Boltzmann thermometry with two excited levels allows precise temperature sensing, but only within a limited temperature range. While low temperatures slow down the nonradiative transitions required to generate a measurable population in the higher excitation level, temperatures that are too high favour equalized populations of the two excited levels, at the expense of low relative thermal sensitivity. In this work, we extend the concept of Boltzmann thermometry to more than two excited levels and provide quantitative guidelines that link the choice of energy gaps between multiple excited states to the performance in different temperature windows. By this approach, it is possible to retain the high relative sensitivity and precision of the temperature measurement over a wide temperature range within the same system. We demonstrate this concept using YAl_3_(BO_3_)_4_ (YAB):Pr^3+^, Gd^3+^ with an excited ^6^P_*J*_ crystal field and spin-orbit split levels of Gd^3+^ in the UV range to avoid a thermal black body background even at the highest temperatures. This phosphor is easily excitable with inexpensive and powerful blue LEDs at 450 nm. Zero-background luminescence thermometry is realized by using blue-to-UV energy transfer upconversion with the Pr^3+^−Gd^3+^ couple upon excitation in the visible range. This method allows us to cover a temperature window between 30 and 800 K.

## Introduction

Chemical reactions, biological functionalities, and various physical phenomena are all strongly determined by the local temperature. The measurement of local temperatures on successively smaller scales requires new methods of temperature sensing that rely on a remote detection principle. Several such concepts already exist. The most commonly known example is infrared (IR) thermography or pyrometry^1–3^, which only allows temperature measurements of surfaces of objects based on their emitted thermal radiation. According to the Stefan−Boltzmann law, the intensity of the emitted radiation scales with the fourth power of temperature and is expected to be sensitive for high temperatures and objects with high emissivity.

An alternative method of remote temperature sensing is luminescence thermometry, which exploits the fact that luminescence properties such as the emission intensity or the luminescence decay time are highly dependent on the local temperature of the surroundings of the luminescent species^4–6^. It requires a simple setup consisting of a laser excitation source, luminescent micro- or nanocrystals or molecules embedded in the object of interest, and a fast and efficient light detection system^7–9^. Its promising advantages over thermography include the lower accessible temperature ranges and non-invasive temperature detection below surfaces. Its successful application in biological media has been highlighted for many different optical centres, such as nitrogen vacancy (NV^−^) centres in fluorescent nanodiamonds^10–12^, semiconducting quantum dots (QDs)^13–15^, bulk halides^16,17^, and organic fluorophores^18–21^.

An alternative emerging class of luminescent thermometers are micro- or nanocrystals doped with trivalent lanthanide ions^22,23^. Their rich electronic level structure that arises from the partly filled 4f^*n*^ (*n* = 1–14) inner shell with characteristic energy gaps on the order of thermal energies (several 100 cm^−1^) allows for thermal coupling between energetically adjacent levels. Most representatives of this class of luminescent thermometers thus rely on ratiometric sensing; in ratiometric sensing, the temperature is measured by means of the intensity ratio of the emission bands from two thermally coupled excited levels. The key advantages of this approach are its simplicity and that it is self-referenced. Moreover, the narrow linewidths of the intraconfigurational 4f^*n*^−4f^*n*^ transitions minimize spectral overlaps between emission lines from two thermally coupled energy levels, even when the energy separation is small. The approximate independence of their energies from the surrounding host material related to the low radial extension of the 4f orbitals allows us to select the emission energy range of interest by appropriately choosing the lanthanide ion. Recently, there has also been progress in reverting this scheme and performing thermometry by detecting a reference emission from the same excited level upon dual excitation from two thermally coupled ground levels^24–26^. Luminescence (nano)thermometry has been demonstrated to be both a precise and accurate technique for probing fundamental thermodynamic phenomena at the micro- and nanoscale^27–29^ and helps to assess the local temperature fluctuations in tissue^30–35^ or in chemical reactors^36–40^. Currently, there has been observable progress on how to implement and standardize this technique for applications^41–45^, the connected limitations^46,47^, or how to couple temperature sensing with other functionalities such as single-molecule magnetism^48,49^ and (meso-)porous materials^50,51^, e.g., theranostics^52^.

The intensity ratio of the emission lines originating from two thermally coupled excited levels within the same configuration follows simple Boltzmann statistics if thermalization is faster than population decay (by any radiative or nonradiative pathways)^53,54^. It has been recently demonstrated that despite their appealing simplicity, Boltzmann-based thermometers with two excited levels suffer from a fundamental thermodynamic limitation^55^. At very low temperatures compared to the energy gap ∆*E*_21_ between the two excited levels |1〉 and |2〉 (*k*_B_*T* ≪ ∆*E*_21_), the probability of thermally exciting the population from the lower to the higher excited emissive level is vanishingly small, which translates to a negligible intensity of the emission from the higher excited level. In addition, the nonradiative absorption rate governing the thermalization from level |1〉 to |2〉 becomes so slow that it can no longer compete with the radiative decay of level |1〉, which leads to a decoupling of the two excited states. In contrast, at very high temperatures compared to the energy gap (*k*_B_*T* ≫ ∆*E*_21_), the populations of the two excited levels in the thermodynamic limit effectively equalize and thus lower the sensitivity of the thermometer, which relies on a relative net change in population between the two excited levels. Both described extremes diminish the overall achievable precision of the thermometer and imply that any Boltzmann-based two-level thermometer only performs with sufficient statistical precision within a limited temperature range dependent on the energy gap ∆*E*_21_ between the two excited levels.

A way to circumvent this thermodynamic limitation and to widen the temperature range for the highest precision of a Boltzmann-based thermometer is to extend the concept of thermal coupling between more than two energetically close excited levels. This method allows us to retain both an optimized response and sensitivity of a single luminescent thermometer by monitoring emission intensity ratios from multiple higher energy excited levels separated by different energy gaps from the lowest excited emissive state. While this has already been realized on a qualitative level^56–58^, no clear guidelines have been established with regard to the most advisable temperature ranges for energy gaps for two or more higher excited levels thus far. Knowledge of the fundamental thermodynamic properties of such a thermally coupled multilevel Boltzmann-based luminescent thermometer helps decide at which temperatures a change to another luminescence intensity ratio (LIR) is advisable to achieve the highest thermometry precision and which combination of energy gaps is optimal for the (wide) temperature range of interest.

Luminescence thermometry at high temperatures also suffers from another practical problem. Any solid emits thermal or black-body radiation, which becomes more intense at high temperatures. Moreover, Wien’s displacement law (*λ*_max_*T* *=* *b*) with *b* ≈ 2.8978 mm∙K) states that the peak wavelength of the Planckian black-body spectrum shifts to shorter wavelengths at high temperatures. While the peak maximum clearly lies in the infrared range at usual lab-accessible temperatures (*T* < 2000 K), the short-wavelength tail of the black-body spectrum can already interfere with the luminescence spectrum in the visible range at temperatures as low as 800 K. Thus, any ratiometric luminescent thermometer devised for high temperature sensing best relies on emission in the ultraviolet range, which is clearly unaffected by black-body background at temperatures below 1200 K.

In this work, we present such a designed multilevel luminescent thermometer that uses the three excited ^6^P_*J*_ (*J* = 7/2, 5/2, 3/2) crystal fields and spin-orbit levels of the UV B-emitting lanthanide ion Gd^3+^; we were motivated by thermodynamic considerations and demonstrated how a single luminescent phosphor can be optimized for precise thermometry from cryogenic (30 K) to high temperatures (800 K) with constantly high relative sensitivities above 0.5% K^−1^. Intense excitation with a cost-effective blue wavelength of 450 nm is interesting for practical applications. This is possible by co-doping with Pr^3+^ in huntite-type crystallizing YAl_3_(BO_3_)_4_ (YAB)^59^ and upconversion into its 4f^1^5d^1^ configuration^60–64^, followed by efficient energy transfer to the ^6^I_*J’*_ (*J’* = 17/2…7/2) levels of Gd^3+^. Not only does this offer the possibility of background free upconversion thermometry, but it also bears potential for applications such as UV lasing^64^ or visible light excited photocatalysis^65^ combined with local temperature sensing. Similar visible-to-UV upconversion relying on triplet-triplet annihilation has otherwise been recently reported by Harada et al. for metal-organic compounds^66^

## Results

### Thermometric performance of an excited three-level system

The benefit of a third emissive level on the performance of a luminescent thermometer can be appreciated as follows. The thermal response (often also referred to as absolute sensitivity), *S*_a_(*T*), of the luminescence intensity ratio (LIR) is directly related to the absolute effective thermal population of the microstate of the higher excited level from which the emission arises. The relative sensitivity, *S*_r_(*T*), in contrast, relates to the relative net change in population between the two thermally coupled excited levels of interest^55^. As indicated above, the thermodynamically optimized performance of a two-excited level thermometer is limited to a small temperature range only. This performance is related to the low response at low temperatures due to the almost negligible thermal population in the higher excited state and a low relative sensitivity at high temperatures due to almost equalized populations (per microstate) in both thermally coupled excited levels. The optimum temperature range depends on the probed energy gap ∆*E*_21_ and is given by^55^

1 $$T_{{{{\rm{opt}}}}} \in \left[ {\frac{{\Delta E_{21}}}{{\left( {2 + \sqrt 2 } \right)k_{{{\rm{B}}}}}},\frac{{\Delta E_{21}}}{{2k_{{{\rm{B}}}}}}} \right]$$

A simple way to widen this optimum temperature range is the addition of a third excited level |3〉 separated by an energy gap ∆*E*_31_ = (1 + *s*)∆*E*_21_ from the lowest excited level |1〉. According to Eq. (1), a widening of the optimum temperature range can be achieved if $s \ge \sqrt 2 /2 \approx 0.707$ and ∆*E*_31_ is used as the thermometric measure above the critical temperature $T^{\prime} = \frac{{\Delta E_{21}}}{{2k_{{{\rm{B}}}}}}$.

**Fig. 1:**
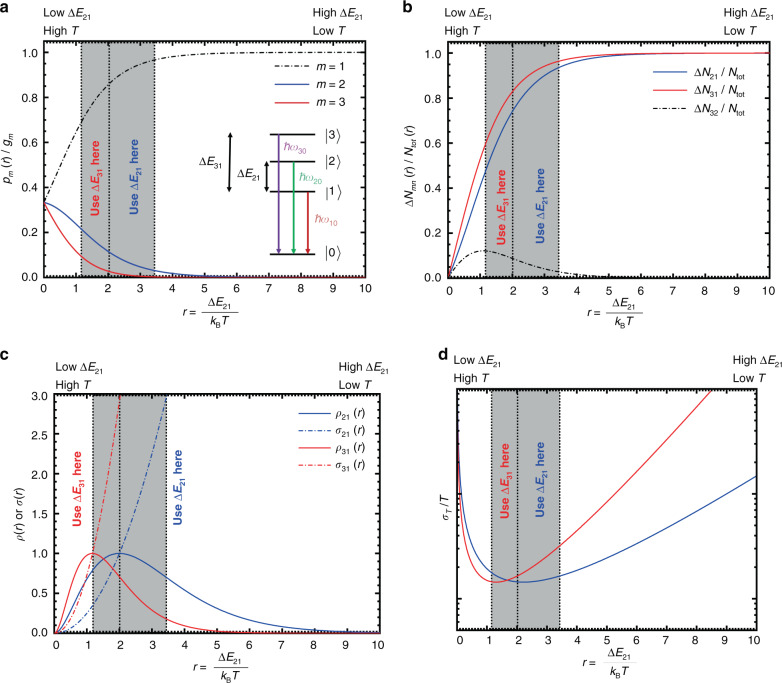
Thermodynamic foundation for the optimum performance of an excited three-level Boltzmann-based luminescent thermometer. All functions are plotted in terms of the dimensionless variable $r = \frac{{\Delta E_{21}}}{{k_{\mathrm{B}}T}}$, and the higher energy gap was exemplarily set to Δ*E*_31_ = 1.71Δ*E*_21_. **a** Plots of the thermal population probabilities per microstate, *p*_*m*_/*g*_*m*_, of an excited three-level system in thermodynamic equilibrium determining the *response* of a luminescent thermometer. *g*_*m*_ denotes the degeneracy of the electronic state |*m*〉. **b** Relative net population changes per microstate, ∆*N*_*mn*_/*N*_tot_, of an excited three-level system in thermodynamic equilibrium determining the *sensitivity* of a luminescent thermometer. Given known ∆*E*_21_ and ∆*E*_31_ values, it is possible to state for which temperature a change in the thermometric measure is advisable. **c** Resulting normalized thermal response functions *ρ*(*r*) (solid) and relative sensitivity functions *σ*(*r*) (dashed-dotted) for thermometry along the energy gaps ∆*E*_21_ (blue) and ∆*E*_31_ (red). **d** Resulting curves for the relative statistical measurement uncertainty, $\sigma _T/T$, of the designed Boltzmann thermometer with excited-state energy gaps ∆*E*_21_ and ∆*E*_31_

This proposal is schematically depicted in Fig. 1a–c. Above $T^{\prime}$, the relative net change in population between levels |3〉 and |1〉 becomes higher than the respective net change between levels |2〉 and |1〉, resulting in a higher relative sensitivity of the thermometer upon the exploitation of the higher energy gap. This strategy can also be generalized to more than three levels in an iterative manner.

### Boltzmann cryothermometry with Gd^3+^

**Fig. 2:**
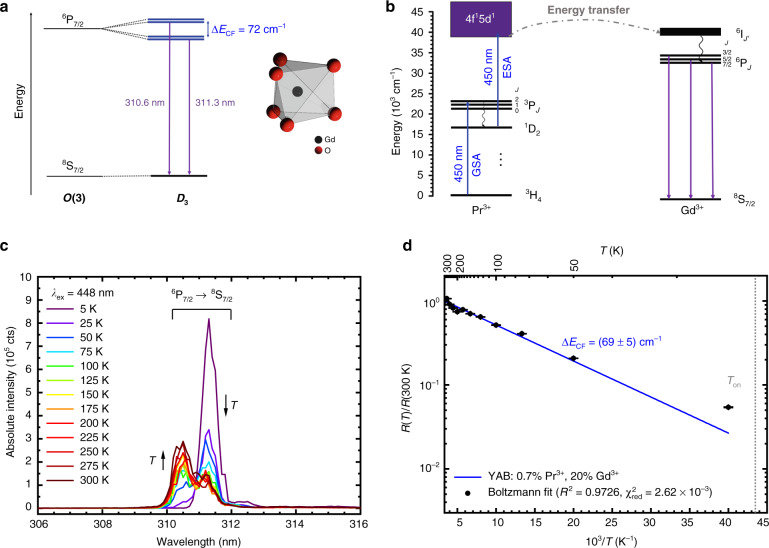
Thermodynamically optimized Boltzmann cryothermometry with Gd^3+^ in YAB:Pr^3+^, Gd^3+^. **a** Splitting of the lowest excited ^6^P_7/2_ spin-orbit level of Gd^3+^ into the different Kramers’ doublets at the present *D*_3_ site symmetry in YAB. The doped Gd^3+^ ions are sixfold coordinated in the form of a twisted trigonal prism (inset). **b** Indirect upconversion excitation scheme for Gd^3+^ by energy transfer from the 4f^1^5d^1^-related electronic states of Pr^3+^. Both the ground state absorption (GSA) and the excited state absorption (ESA) of two 450 nm photons within the pulse period (*T* = 50 ms) of the used pulsed laser source are indicated. **c** High-resolution upconversion photoluminescence spectra of YAB: 0.7% Pr^3+^, 20% Gd^3+^ upon excitation of Pr^3+^ at 448 nm showing the temperature dependence of the two radiative transitions from the lower and higher crystal field states of the ^6^P_7/2_ level. The arrows mark the two thermometrically employed transitions. **d** Boltzmann plot of the temperature-dependent LIR normalized to its value at 300 K. The fitted energy gap, statistical figures of merit, and expected onset temperature for Boltzmann behaviour (see Eq. (5)) are indicated

The previously introduced thermodynamic concept of a multilevel thermometer can be realized with Gd^3+^. Here, we use the small energy splitting between different crystal field components of the ^6^P_7/2_ level for temperature sensing in the low-temperature regime. In the next section, the larger splitting between ^6^P_7/2_ and higher energetic spin-orbit levels ^6^P_5/2_ and ^6^P_3/2_ for accurate measurements at higher temperatures is exploited instead. A schematic energy level diagram illustrating the working principle of crystal field component-based Boltzmann cryothermometry with Gd^3+^ is depicted in Fig. 2a. For cryogenic temperatures (4 K < *T* < 100 K), Eq. (1) suggests that the most suitable energy gap for the performance of Boltzmann thermometry is on the order of 50−100 cm^−1^. This energy range is typical for the splitting of 4f^n^-based spin-orbit levels by the surrounding crystal field potential, although other examples, such as the *R* lines of Cr^3+^ doped into hosts with strong crystal fields, also have energy differences on this order of magnitude^67–69^.

Gd^3+^ is a very weak absorber^70,71^. In addition, efficient and intense UV light sources are not readily available, and direct UV excitation also gives rise to background emission. We thus chose a different strategy by co-doping the sample with Pr^3+^, which is schematically depicted in Fig. 2b. Upon excitation with a pulsed optical parametric oscillator (OPO) in the blue range at 448 nm, the Pr^3+^ ions are excited to the ^3^P_2_ level. After quick temperature-independent nonradiative relaxation to the ^1^D_2_ level given the high phonon energies in YAB ($\hbar \omega _{\max }\sim 1300\,{{{\rm{cm}}}}^{ - 1}$)^72^, electric dipole-allowed excited state absorption (ESA) into the 4f^1^5d^1^ configuration of Pr^3+^ at energies in the range of 40,000 cm^−1^ can take place^72^, which is followed by an energy transfer to the ^6^I_*J’*_ levels of Gd^3+^ at energies of approximately 37000 cm^−1^ (270 nm). The dependence of the Gd^3+^-related emission intensity on the incident power of the OPO also indicates a two-photon upconversion process (see Supplementary Information, Fig. S1). Similar upconversion spectra were, however, also measured using an inexpensive continuous wave (CW) blue laser with 1 W output power (see Supplementary Information, Fig. S2), which demonstrates that this concept also works with any high-power LED.

Since Pr^3+^ is very prone to the resonant cross-relaxation process [Pr1, Pr2]: [^3^P_0_, ^3^H_4_] → [^1^G_4_, ^1^G_4_], it was mandatory to keep the Pr^3+^ concentration at only 0.7 mol% (see also Supplementary Information, Fig. S3). The large energy gap between the ^6^P_*J*_ (*J* = 7/2…3/2) levels and the ^8^S_7/2_ ground level of Gd^3+^, which does not have any intermediate electronic levels, as well as the high mutual lanthanide distances in the YAB host structure (~ 5.9 Å)^59^, allow an increase in the Gd^3+^ concentration to 20 mol% without any observable concentration quenching effects (see Supplementary Information, Fig. S4). The high energy gap increases the probability of efficient energy transfer from Pr^3+^. Fig. 2c depicts a portion of the luminescence spectra of YAB: 0.7% Pr^3+^ and 20% Gd^3+^ appear in the UV-B range and clearly show that the strategy to excite Gd^3+^ via Pr^3+^-based upconversion and subsequent energy transfer does work. The absence of a 4f^1^5d^1^ → 4f^2^ broad-band emission of Pr^3+^ also shows that the energy transfer from Pr^3+^ to Gd^3+^ must be very efficient, which is in agreement with earlier findings^73^.

Boltzmann thermometry with Gd^3+^ at cryogenic temperatures becomes possible if the two clearly resolved components of the ^6^P_7/2_ → ^8^S_7/2_-based group of radiative transitions are chosen (see Fig. 2a). These transitions give rise to two intense emission peaks located at 310.6 nm and 311.3 nm (see Fig. 2b), whose intensity ratio (higher energetic to lower energetic emission) is used as the temperature-correlated signal. The LIR at temperature *T, R*(*T*), normalized to its value at the highest measured reference temperature, *T*_0_, thus follows the law^23,74^

2 $$\frac{{R\left( T \right)}}{{R\left( {T_0} \right)}} = \exp \left[ { - \frac{{\Delta E_{m1}}}{{k_{{{\rm{B}}}}}}\left( {\frac{1}{T} - \frac{1}{{T_0}}} \right)} \right]$$

where *k*_B_ is the Boltzmann constant and ∆*E*_*m*1_ (*m* > 1) is the energy gap between the two thermally coupled levels and is the only fitting parameter. A fit to a linearized version of Eq. (2) gave an effective energy gap of ∆*E*_CF_ = (69 ± 5) cm^−1^, in excellent agreement with the value of 72 cm^−1^ determined from the emission spectra. It should be noted that a corresponding splitting of the ^8^S_7/2_ level into Kramers’ doublets is much smaller (on the order of only 1 cm^−1^) if spin-orbit coupling is only intermediate because of the lack of orbital degeneracy given the quantum number *L* = 0^75^. This makes the ^8^S_7/2_ ground level an effective single level in the case of Gd^3+^.

The LIR deviates from the expected Boltzmann behaviour at temperatures below 25 K. This observation is related to a kinetic limitation. The nonradiative absorption rate from the lower to the higher excited level, $k_{{{{\rm{nr}}}}}^{{{{\rm{abs}}}}}\left( T \right)$, is given by^76,77^

3 $$k_{{{{\rm{nr}}}}}^{{{{\rm{abs}}}}}\left( T \right) = g_mk_{{{{\rm{nr}}}}}\left( 0 \right){\langle{n}\rangle}^p$$

with *g*_*m*_ as the degeneracy of the higher excited level (*g*_*m*_ = 2 for Kramers’ doublets), *k*_nr_(0) as an intrinsic nonradiative rate governed by the properties of the electronic transition, and

4 $$\left\langle n \right\rangle = \frac{1}{{\exp \left( {\frac{{\hbar \omega _{{{{\rm{ph}}}}}}}{{k_{{{\rm{B}}}}T}}} \right) - 1}}$$

as the thermally averaged phonon occupation number of an acoustic or optical phonon mode with energy $\hbar \omega _{{{{\rm{ph}}}}}$ and *p* as the number of phonons consumed during the thermalization between the two excited levels (∆*E*_*m*1_ = $p\hbar \omega _{{{{\rm{ph}}}}}$, here it is *p* = 1). This temperature-dependent nonradiative absorption rate competes with the radiative decay (and eventually other quenching) rates from level |1〉. At sufficiently low temperatures, the nonradiative absorption rate decreases than the intrinsic radiative decay rate, and thus, Boltzmann thermalization is kinetically inhibited. We have recently shown that a very accurate estimate for the expected onset temperature of Boltzmann behaviour, *T*_on_, is given by^55^

5 $$T_{{{{\rm{on}}}}} = 0.2227\frac{{\Delta E_{m1}}}{{k_{{{\rm{B}}}}}}$$

With the spectroscopic value of ∆*E*_CF_ = 72 cm^−1^, the expected onset temperature for Boltzmann thermalization between the two excited levels is thus approximately 23 K, very close to the observed deviation from Boltzmann behaviour below 25 K. A similar deviation has also been observed in the case of Cr^3+^^67,78^, which can be explained by the same kinetic effect. In turn, the optimum temperature range for the most precise thermometry that exploits this energy gap between the two Kramers’ doublets of the ^6^P_7/2_ level of Gd^3+^ is between 30 and 51 K according to Eq. (1). The connected relative sensitivities, *S*_r_(*T*), as defined by

6 $$S_r\left( T \right) = \left| {\frac{1}{{R\left( T \right)}}\frac{{{{{\rm{d}}}}R}}{{{{{\rm{d}}}}T}}} \right| = \frac{{\Delta E_{m1}}}{{k_{{{\rm{B}}}}T^2}}$$

in the case of a Boltzmann thermometer that varies between *S*_r_(30 K) = 11.6% K^−1^ and *S*_r_(51 K) = 3.98% K^−1^ in this optimum range, which is clearly above the usually desired threshold of *S*_r_ = 1% K^−1^ in practice^23^. In fact, the relative sensitivity at 30 K is the highest reported value for luminescent cryothermometers at that particular temperature thus far^50,67,68,79,80^. It is important to note, however, that it is both the balance between reliably detectable luminescence signals and relative sensitivity that determine the overall precision of a luminescent thermometer (see also below)^55^^,^^81^. Another important point is that for accurate cryothermometry with small energy gaps, a high spectral resolution spectrometer is also required to separate two emission lines that are close in wavelength. Finally, that high relative sensitivities at cryogenic temperatures are not surprising, as can already be appreciated from the *T*^−2^ dependence in Eq. (6) that dominates at low temperatures and stems from the strong relative net change in population between the two thermally coupled excited levels in this domain (see Fig. 1b).

### Extension of high-precision thermometry with Gd^3+^ to higher temperatures

**Fig. 3:**
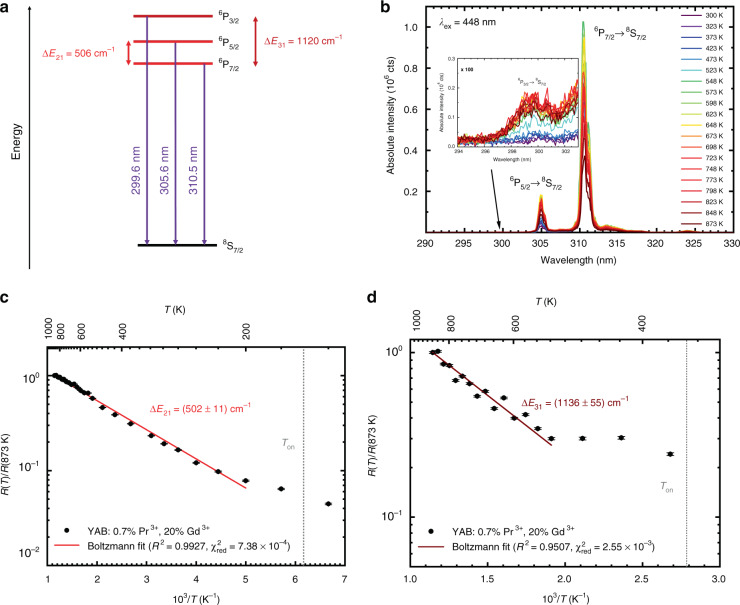
Boltzmann thermometry was thermodynamically optimized at room temperature and higher temperatures (*T* > 500 K) with Gd^3+^ in YAB:Pr^3+^, Gd^3+^. **a** Spin-orbit level scheme and respective radiative transitions depicting the concept for high-precision Boltzmann thermometry with Gd^3+^. **b** High-resolution upconversion photoluminescence spectra of YAB: 0.7% Pr^3+^, 20% Gd^3+^ upon excitation of Pr^3+^ at 448 nm showing the radiative transitions from the different ^6^P_*J*_ levels (*J* = 7/2, 5/2, and 3/2) of Gd^3+^. The spectra were normalized with respect to the highest intensity peak (*I*_10_ ~ 10^6^ counts) to demonstrate the thermally induced intensity increase of the higher energetic emission peaks. **c** Boltzmann plot of the temperature-dependent LIR employing the transitions from the ^6^P_5/2_ and ^6^P_7/2_ levels, respectively, normalized to its value at 873 K. The fitted energy gap, statistical figures of merit and expected onset temperature for Boltzmann behaviour (Eq. (5)) are indicated. **d** Boltzmann plot of the temperature-dependent LIR, which employs the transitions from the ^6^P_3/2_ and ^6^P_7/2_ levels, normalized to its value at 873 K. The fitted energy gap, statistical figures of merit, and expected onset temperature for Boltzmann behaviour (Eq. (5)) are indicated

The small crystal field splitting between the Kramers’ doublets stemming from the ^6^P_7/2_ spin-orbit level of Gd^3+^ is not well suited for temperature measurements above 100 K since the excited state populations in the two thermally coupled crystal field states equalize at elevated temperatures. This fact in turn lowers both the response and sensitivity of the chosen radiative transitions for temperature sensing. For thermometry in the range of room temperature, it is necessary to employ two levels with a higher energy gap. According to Eq. (1), a suitable energy gap to measure temperatures between 200 and 400 K is on the order of 500–700 cm^−1^, in the range of spin-orbit interaction-induced splitting for the 4f^n^-based electronic levels of lanthanides. Consequently, we used the LIR of the radiative transitions from the ^6^P_7/2_ and ^6^P_5/2_ spin-orbit levels to the ^8^S_7/2_ ground level (see Fig. 3a) of Gd^3+^ to demonstrate its applicability for temperature sensing in the range of room temperature (see Fig. 3b). The LIR of the ^6^P_5/2_ to ^6^P_7/2_ emission lines was measured as a function of temperature as determined from the emission spectra shown in Fig. 3b and fit to Eq. (2) (Fig. 3c). The results indicated an effective energy gap between the ^6^P_7/2_ and ^6^P_5/2_ levels of ∆*E*_21_ = (502 ± 11) cm^−1^, again in excellent agreement with the value of 506 cm^−1^ that was spectroscopically deduced from the emission spectra. With this energy gap, the most suitable temperature range for high-precision luminescence thermometry is between 213 and 364 K with corresponding relative sensitivities of *S*_r_(213 K) = 1.60% K^−1^ and *S*_r_(364 K) = 0.55% K^−1^. These relative sensitivities are almost one order of magnitude higher than the corresponding sensitivities for thermometry with crystal field splitting of 72 cm^−1^ in this temperature range. This result demonstrates that the concept of multiple excited states with different energy separations could realize high-temperature sensing accuracy over a wider temperature range, given sufficiently high photon counts of the emission bands of interest. As in the case of cryothermometry, the LIR deviates from Boltzmann behaviour below 200 K, which is a consequence of the very slow nonradiative absorption in that temperature range^53–55^. This observation also coincides very well with the estimated onset temperature for Boltzmann behaviour at *T*_on_ = 163 K according to Eq. (5) for an energy gap of 506 cm^−1^.

An extension of the concept of optimized luminescence thermometry with Gd^3+^ to even higher temperatures is finally possible by the exploitation of thermal coupling between the excited ^6^P_3/2_ and ^6^P_7/2_ levels (Fig. 3d). The corresponding LIR is clearly only a useful temperature measure at very high temperatures due to the generally low intensity of the ^6^P_3/2_ → ^8^S_7/2_-based transition located at 295 nm (inset of Fig. 3b). In particular, this application also demonstrates why Gd^3+^ is a particularly useful choice as a luminescent thermometer for high-temperature sensing. Since there are no lower-lying energy levels between the ^6^P_7/2_ level located approximately 32000 cm^−1^ above the ^8^S_7/2_ ground state, thermal quenching of the Gd^3+^-based luminescence in a wide bandgap host compound such as YAB cannot take place. Fitting the temperature dependence of the corresponding LIR that stems from the two excited levels ^6^P_3/2_ and ^6^P_7/2_ to Eq. (2) (Fig. 3d) gives an effective energy gap of ∆*E*_31_ = (1136 ± 81) cm^−1^, again in excellent agreement with the spectroscopically determined value of 1120 cm^−1^. This energy gap is optimally suited to measure temperatures between 472 and 805 K with corresponding relative sensitivities of *S*_r_(472 K) = 0.73% K^−1^ and *S*_r_(805 K) = 0.25% K^−1^, which is an improvement by a factor of 2.2 compared to thermometry with the ^6^P_5/2_−^6^P_7/2_ gap in this temperature range. Here, again, thermalization and the sustainment of a Boltzmann equilibrium between the ^6^P_3/2_ and ^6^P_7/2_ spin-orbit levels of Gd^3+^ is only observed above temperatures of at least 450 K, while Eq. (5) suggests an expected onset temperature of approximately 360 K. The discrepancy between observed and estimated onset is related to the weak intensity of the ^6^P_3/2_ → ^8^S_7/2_ transition that makes it difficult to accurately determine the LIR below 450 K.

**Fig. 4:**
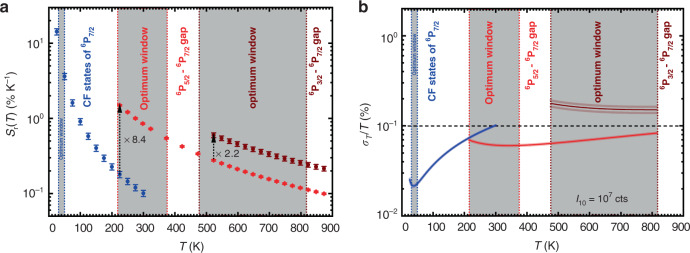
Assessment of the optimum performance of a wide-range multilevel luminescent Boltzmann thermometer based on Gd^3+^ in YAB:Pr^3+^, Gd^3+^. **a** Plot of the relative sensitivities *S*_r_ of the different temperature measures and their improvement upon thermodynamically guided change of the respective energy gap of the excited levels of Gd^3+^ used for temperature sensing. **b** Overall theoretical relative temperature uncertainty of the different energy gaps assuming a constant integrated intensity measure of *I*_10_ = 10^7^ counts of the selected lowest energetic emission. A relative temperature uncertainty of 0.1% is desirable because it allows the measurement of temperatures below 1000 K with a statistical error of ±1 K. The shaded areas indicate the statistical fluctuations in the relative temperature uncertainty

The photoluminescence spectra reveal that the intensity of the radiative emission from the ^6^P_3/2_ spin-orbit level of Gd^3+^ is very weak compared to the emission from the ^6^P_7/2_ level. According to calculations by Detrio on Gd^3+^, the ^6^P_3/2_ → ^8^S_7/2_ transition is generally characterized by very low intensities irrespective of the chosen host material given the very low reduced matrix elements governing the intensities of dipole-allowed electronic transitions in Judd−Ofelt theory^82^. This trend raises the question of whether the LIR between the radiative transitions from the thermally coupled ^6^P_3/2_ and ^6^P_7/2_ levels actually offers a more precise temperature measure based on the higher relative sensitivity *S*_r_(*T*) or whether continuous exploitation of the LIR between the transitions from the ^6^P_5/2_ and ^6^P_7/2_ levels up to higher temperatures is more favourable based on the higher luminescence intensity of the ^6^P_5/2_ → ^8^S_7/2_ radiative transition. For that purpose, we calculated the minimum theoretical relative temperature uncertainty for both thermometry measures within the optimum temperature range of the ^6^P_3/2_−^6^P_7/2_ gap (472−805 K). The relative temperature precision of a ratiometric Boltzmann-based thermometer is given by^55^

7 $$\frac{{\sigma _T}}{T} = \frac{{k_{{{\rm{B}}}}T}}{{\Delta E_{m1}}}\frac{1}{{\sqrt {I_{10}} }}\sqrt {1 + \frac{1}{{R\left( T \right)}}} = \frac{{k_{{{\rm{B}}}}T}}{{\Delta E_{m1}}}\frac{1}{{\sqrt {I_{10}} }}\sqrt {1 + \frac{1}{{R\left( \infty \right)}}\exp \left( {\frac{{\Delta E_{m1}}}{{k_{{{\rm{B}}}}T}}} \right)}$$

where *I*_10_ denotes the intensity (in integrated photon counts) of the lower energetic emission and $R\left( \infty \right)$ is the extrapolated LIR at infinite temperatures that can be obtained from the respective Boltzmann fits. Graphs depicting the evolution of both the relative sensitivity and the relative temperature uncertainty at different temperatures are shown in Fig. 4a, b. They clearly indicate that upon setting the same photon count number of *I*_10_ = 10^7^ counts of the ^6^P_7/2_ → ^8^S_7/2_-based emission, the LIR between the radiative transitions from ^6^P_5/2_ and ^6^P_7/2_ still gives rise to a lower relative temperature uncertainty (ca. 0.08%) based on the higher relative intensity of the ^6^P_5/2_ → ^8^S_7/2_ transition than the corresponding LIR involving the radiative transition from the higher energetic ^6^P_3/2_ level (relative temperature uncertainty of ca. 0.2%). In that case, it is advisable to still retain the LIR stemming from the adjacent ^6^P_5/2_ and ^6^P_7/2_ levels. Thus, it depends on the trade-off between practically achievable intensities and thermodynamically guided optimum temperature range, which is the more advisable choice as a temperature measure to probe high temperatures with Gd^3+^ in YAB: Pr^3+^, Gd^3+^. Irrespective of the choice, it is possible to keep the absolute temperature uncertainty below a threshold of 1.3 K even at 820 K. We do stress, however, that these relative temperature uncertainties are purely statistical and do not include any additional systematic error that could occur in a real-case application. It is also important that these low uncertainties require a high integrated intensity (~10^7^ cts) of the ^6^P_7/2_ → ^8^S_7/2_-related emission of Gd^3+^, which may be difficult to achieve in an application based on the proposed upconversion.

## Discussion

Single ion luminescence thermometry based on a Boltzmann equilibrium between two emitting excited levels is both thermodynamically and kinetically limited to work with optimized precision only within a certain temperature range. If a luminescent thermometer is supposed to cover wider temperature ranges, thermal coupling between more than two excited states must be exploited. In this work, we offer quantitative guidelines based on a recently developed theoretical framework that allows us to extend the usability of a Boltzmann-based luminescent thermometer over wider temperature ranges by carefully choosing the energy gaps between multiple thermally coupled levels. We demonstrate the concept practically for the case of Gd^3+^, which exhibits three energetically well-isolated ^6^P_*J*_ (*J* = 7/2, 5/2, 3/2) levels in the UV range that can additionally rely on splitting in a local crystal field. Gd^3+^ is thus a prime choice for especially high-temperature ratiometric luminescence thermometry, as the black-body background does not interfere in this emission range. Moreover, it absorbs and emits in the solar-blind spectral range, and thus, any background signal from ambient sunlight is also avoided. Since Gd^3+^ is a weak absorber, we demonstrated an alternative excitation scheme with a cost-effective and well-established excitation wavelength of 450 nm available from blue LEDs via Pr^3+^ in a blue-to-UV upconversion process. By ground state absorption into the ^3^P_2_ level, followed by efficient excited-state absorption into the 4f^1^5d^1^ configuration of Pr^3+^, it is possible to indirectly excite Gd^3+^ by energy transfer into its ^6^I_*J’*_ (*J’* = 17/2…7/2) levels, from which the excited state population finally undergoes nonradiative relaxation into the ^6^P_*J*_ levels. Optimized Boltzmann cryothermometry between 30 and 50 K was possible with two crystal field components of the excited ^6^P_7/2_ level separated by 72 cm^−1^. Temperature sensing in the range of room temperature is better performed by exploiting the LIR of the radiative transitions stemming from the ^6^P_5/2_ and ^6^P_7/2_ spin-orbit levels of Gd^3+^ separated by 506 cm^−1^. Finally, high-temperature thermometry between 470 and 800 K is also possible using Boltzmann thermalization between the ^6^P_3/2_ and ^6^P_7/2_ levels of Gd^3+^, with an energy separation of 1120 cm^−1^. Both the ^6^P_3/2_ → ^8^S_7/2_- and the ^6^P_5/2_ → ^8^S_7/2_-based emissions were compared in terms of the overall achievable temperature precision of the respective ratiometric thermometry approaches. While the ^6^P_3/2_ → ^8^S_7/2_-based emission is very low in intensity, it offers the necessary high relative sensitivity at high temperatures. It was shown that the final choice for high-temperature thermometry with Gd^3+^ depends on the practically achievable integrated intensity of the lowest energetic ^6^P_7/2_ → ^8^S_7/2_ transition.

In summary, a theoretical framework is presented to quantitatively understand and design single ion thermometers relying on multiple thermally coupled excited states to widen the window for accurate thermal sensing. The concept is shown to be feasible using a Pr^3+^, Gd^3+^-coactivated YAl_3_(BO_3_)_4_ phosphor with Gd^3+^ as a Boltzmann-based single ion luminescent thermometer. UV upconversion emission from the ^6^P_*J*_ levels of Gd^3+^ is sensitized via two-step excitation of Pr^3+^ at 450 nm to the states of the 4f^1^5d^1^ configuration, followed by energy transfer to Gd^3+^, which is practically feasible with the use of high-power blue LEDs. Analysis of the temperature-dependent emission demonstrates how simple thermodynamic principles can be applied to circumvent the fundamental limitations in temperature precision of a conventional two-level single ion luminescent thermometer to finally make one thermometry system applicable to a wide range of temperatures while retaining high precision. This work paves the way for the targeted design of luminescent thermometers tailored towards applicational requirements for temperature accuracy within specific temperature ranges.

## Methods

### Chemical reagents

Y(NO_3_)_3_ ∙ 6 H_2_O (Alfa Aesar, Germany, 99.99%), Al(NO_3_)_3_ ∙ 9 H_2_O (Sigma–Aldrich, Germany, ≥ 98%), Pr(NO_3_)_3_ ∙ 6 H_2_O (Strem Chemicals, France, 99.9%), Gd(NO_3_)_3_ ∙ 6 H_2_O (Sigma–Aldrich, Germany, 99.99%), H_3_BO_3_ (Merck, Germany, 99.8%) and urea (Sigma–Aldrich, Germany, ≥ 98%) were used without further purification.

### Synthesis of YAl_3_(BO_3_)_4_ (YAB): *x*% Pr^3+^, *y*% Gd^3+^ microcrystalline powder

Microcrystalline powder samples of YAB: *x*% Pr^3+^, *y*% Gd^3+^ (*x* = 0.1, 0.3, 0.5, 0.7, 1.0, and 2.0; *y* = 5, 10, 20, 40, and 60) were prepared by a modified urea–nitrate solution-based combustion route^83^. Stoichiometric amounts of Ln(NO_3_)_3_ ∙ 6 H_2_O (Ln = Y, Pr, and Gd) and Al(NO_3_)_3_ ∙ 9 H_2_O were dissolved in distilled H_2_O (approximately 40 ml) under vigorous stirring at room temperature. Subsequently, solid urea (molar ratio urea/lanthanide ions = 3:1) and H_3_BO_3_ (5 mol% excess) were added to the transparent nitrate-containing solution. The solution was heated to 80 °C to improve the solubility of the H_3_BO_3_ and constantly stirred for 30 min at that temperature. The beaker was covered with parafilm to prevent excessive solvent evaporation. The resulting solution was quickly transferred to an alumina crucible and placed into a preheated furnace at 500 °C in air for 10 min to initiate combustion. The thus-formed colourless solid precursor was carefully ground with additional solid H_3_BO_3_ (half of the previous stoichiometric amount) in a mortar to account for the losses that occurred during the combustion step. The solid mixture was finally sintered at 1100 °C for 6 h in air. After naturally cooling to room temperature, the obtained colourless residue was ground to a fine powder, and its phase purity was verified by powder X-ray diffraction (Philips PW391, Cu *K*_*α*1_ radiation (*λ* = 1.54056 Å), *U* = 40 kV, *I* = 20 mA, reflection mode). The X-ray diffraction pattern was scanned in a 2*θ* range between 10° and 80° with a step size of 0.02° (see Supplementary Information, Fig. S5). Morphology and energy dispersive X-ray spectroscopy characterizations were performed using scanning electron microscopy (SEM; Nova, NANO SEM 430; see Supplementary Information, Fig. S6).

### Optical spectroscopy and temperature-dependent measurements

Excitation was performed with an external pulsed wavelength tuneable Opotek Opolette HE 355 II (Carlsbad, CA, USA) OPO pumped by a frequency-tripled Nd:YAG laser at a repetition rate of 20 Hz and a temporal pulse width of approximately 6 ns. Emission spectra were acquired on an Edinburgh FLS920 spectrofluorometer (Livingston, UK) equipped with a 0.25 m double Littrow-configuration grating monochromator blazed at 300 nm and a Hamamatsu R928 (Shizuoka, Japan) photomultiplier tube (PMT) for photon detection. All emission spectra were corrected for grating efficiency and detector sensitivity. Temperature-dependent measurements below room temperature were performed with an Oxford Instruments liquid He flow cryostat (Oxford, UK) and an external temperature control unit, which measured the temperature by means of a thermocouple in direct contact with the powder sample holder. High-temperature luminescence spectra were acquired by placing the sample into an externally water-cooled Linkam (Surrey, UK) THMS600 microscope stage (±0.1 °C temperature stability). The temperature was externally controlled by a thermocouple in immediate contact with the sample holder. Photoluminescence decay curves were acquired by pulsed excitation with the OPO and detection of the time-resolved signal with a multichannel scaler (MCS) attached to a Hamamatsu H7422 PMT (Shizuoka, Japan) for minimized background. For the demonstration of the excitation of the upconverted emission of Gd^3+^ with blue light, a CW laser of 450 nm and maximum output power of 1 W (Changchun New Industries Optoelectronics Technology, Changchun, China) was employed as an excitation source.

## Supplementary information

Supplemental Material
