# Supplementary material for: One ion to catch them all: Targeted high-precision Boltzmann thermometry over a wide temperature range with Gd3+
Source: Light Sci Appl. 2021 Nov 22;10:236. doi: 10.1038/s41377-021-00677-5 (PMC8608900; doi:10.1038/s41377-021-00677-5)
Supplement: Supplementary file 1 — Supplemental Material [file 41377_2021_677_MOESM1_ESM.docx]

Supplementary Information for

One ion to catch them all: Targeted high-precision Boltzmann thermometry over a wide temperature range with Gd^3+^

Dechao Yu,^[a,b]^ Huaiyong Li,^[b,c]^ Dawei Zhang,^[a]^ Qinyuan Zhang,^*[d]^ Andries Meijerink,^[b]^ and Markus Suta^*[b,e]^

[a] *Engineering Research Center of Optical Instrument and System, The Ministry of Education, Shanghai Key Laboratory of Modern Optical System, University of Shanghai for Science and Technology, Shanghai 200093, People’s Republic of China*

[b] *Condensed Matter and Interfaces, Debye Institute for Nanomaterials Science, Department of Chemistry, Utrecht University, Princetonplein 1, 3584 CC Utrecht, The Netherlands*

[c] *School of Materials Science and Engineering, Liaocheng University, Liaocheng 252059, People’s Republic of China*

[d] *State Key Laboratory of Luminescent Materials and Devices, and Institute of Optical Communication Materials, South China University of Technology, Guangzhou 510641, People’s Republic of China*

[e] *Inorganic Photoactive Materials, Institute of Inorganic Chemistry, Heinrich Heine University Düsseldorf, Universitätsstraße 1, 40225 Düsseldorf, Germany*

*Corresponding authors: [qyzhang@scut.edu.cn](mailto:qyzhang@scut.edu.cn); [m.suta@uu.nl](mailto:m.suta@uu.nl); [markus.suta@hhu.de](mailto:markus.suta@hhu.de)


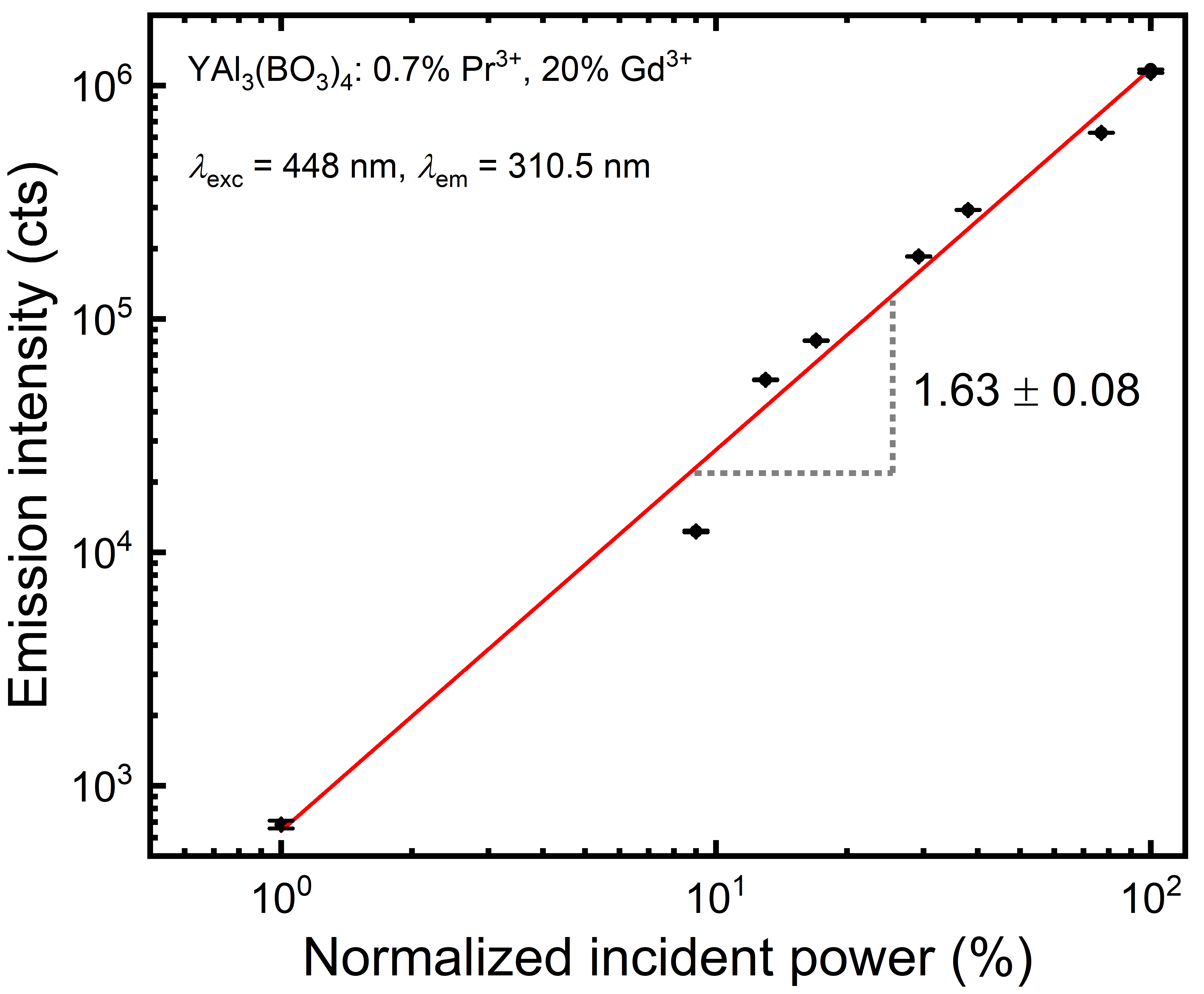


Figure S1. Double log plot of the intensity of the UV emission of Gd^3+^ in YAl_3_(BO_3_)_4_: 0.7% Pr^3+^, 20% Gd^3+^ against the incident OPO laser power (normalized with respect to its maximum output with neutral density filters) indicating an approximate two-photon upconversion process. All spectra were acquired at room temperature.


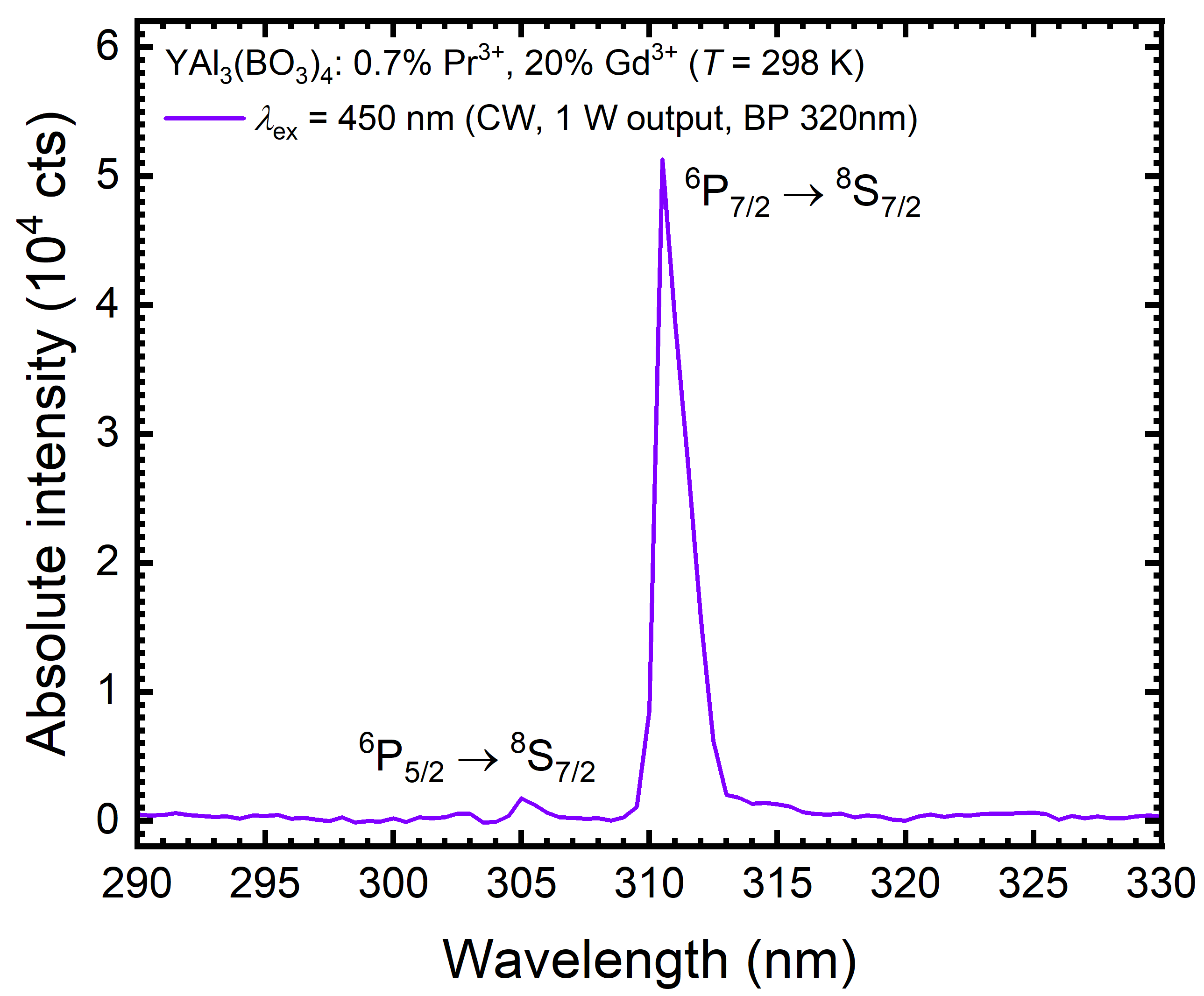


Figure S2. UV upconversion emission spectrum of YAl_3_(BO_3_)_4_: 0.7% Pr^3+^, 20% Gd^3+^ at room temperature upon excitation with a continuous wave (CW) blue laser at 450 nm. The output power was 1 W and a bandpass filter transmitting at 320 nm was placed in front of the sample. The laser beam was not focused on the sample.


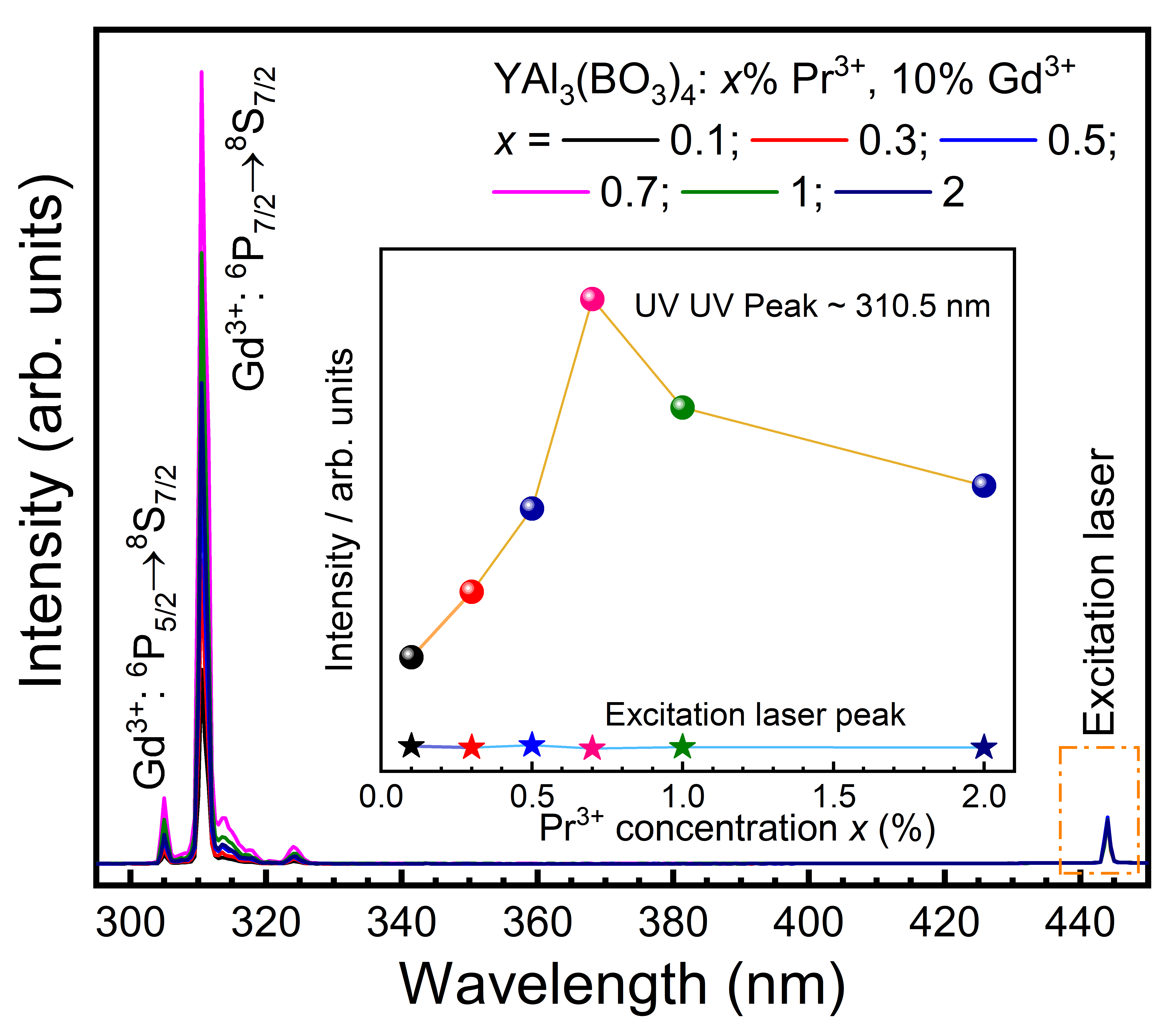


Figure S3. Upconversion emission spectra of YAB: *x*% Pr^3+^, 10% Gd^3+^ upon laser excitation with 448 nm at room temperature. The inset depicts the integrated intensity of the Gd^3+^-based emission at 310.5 nm relative to the excitation scattering peak as an internal intensity reference.


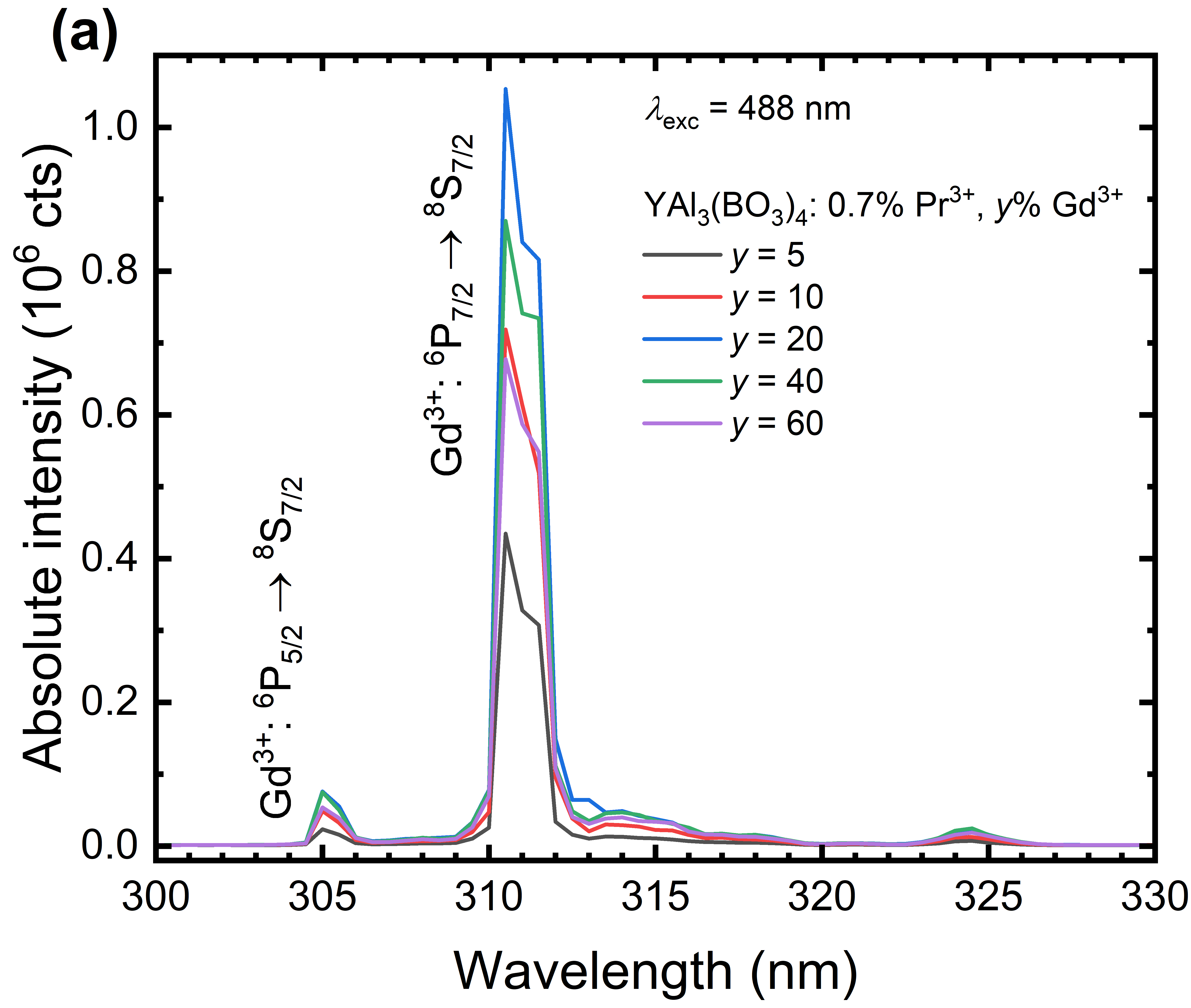

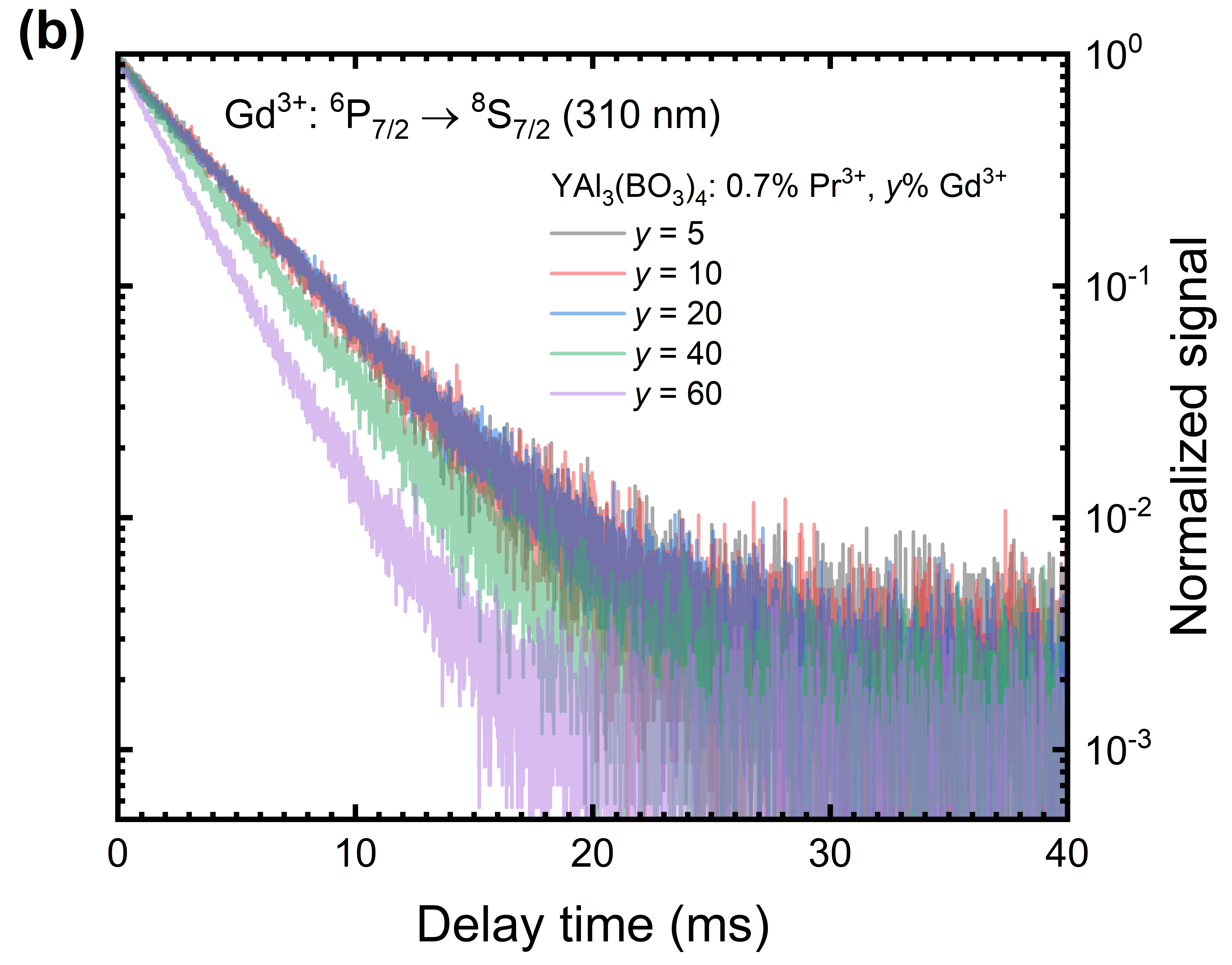


Figure S4. (a) Upconversion emission spectra of YAB: 0.7% Pr^3+^, *y*% Gd^3+^ upon laser excitation with 448 nm at room temperature. (b) Luminescence decay curves of YAB: 0.7% Pr^3+^, *y*% Gd^3+^ upon excitation with 448 nm and monitoring of the emission at 310 nm at room temperature to investigate the impact of concentration quenching on the Gd^3+^-based emission.


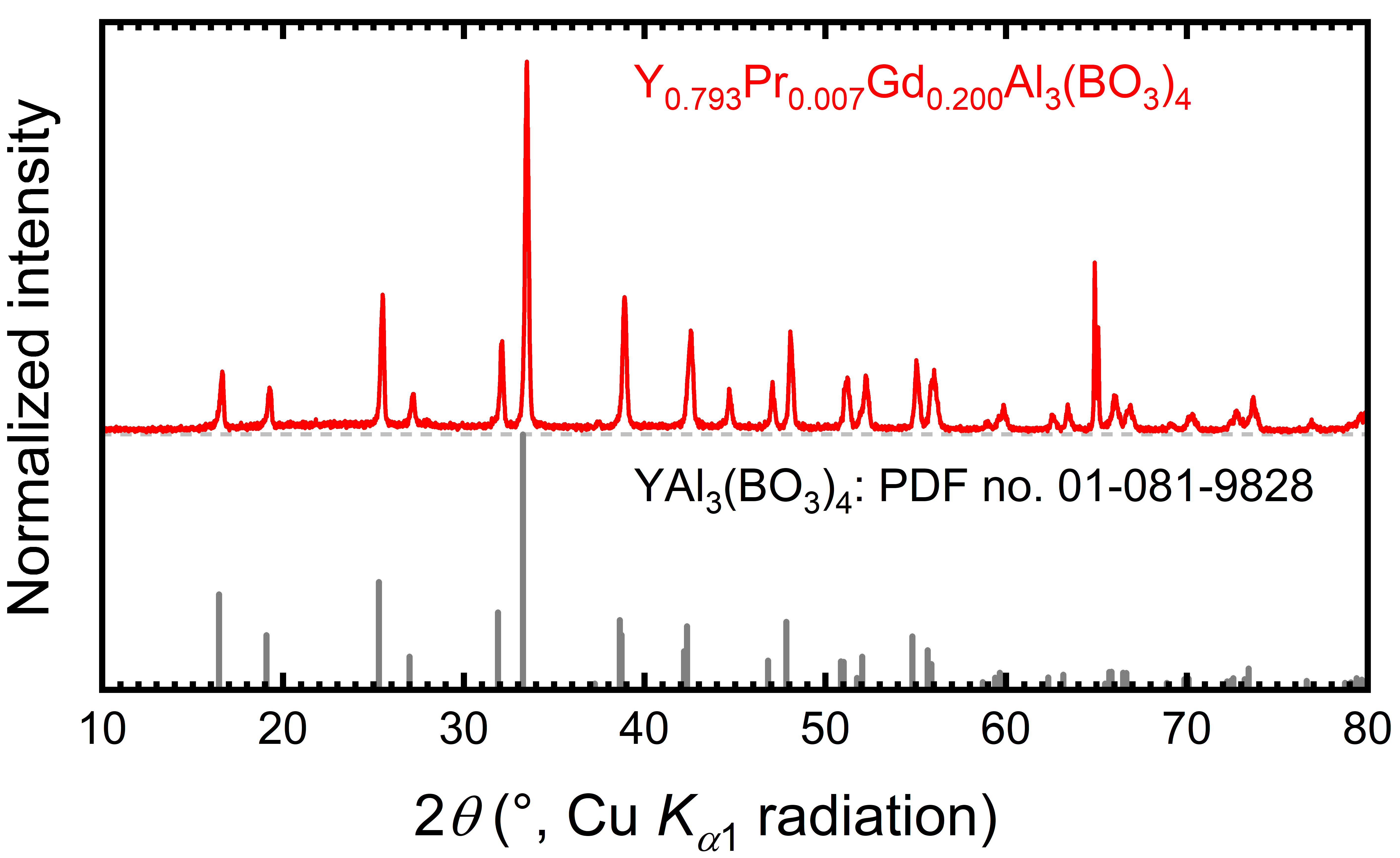


Figure S5. Powder X-ray diffraction pattern (Cu *K_α_*_1_ radiation) of the synthesized microcrystalline YAl_3_(BO_3_)_4_: 0.7% Pr^3+^, 20% Gd^3+^ (YAB: 0.7% Pr^3+^, 20% Gd^3+^) obtained by a urea-nitrate-based combustion synthesis.


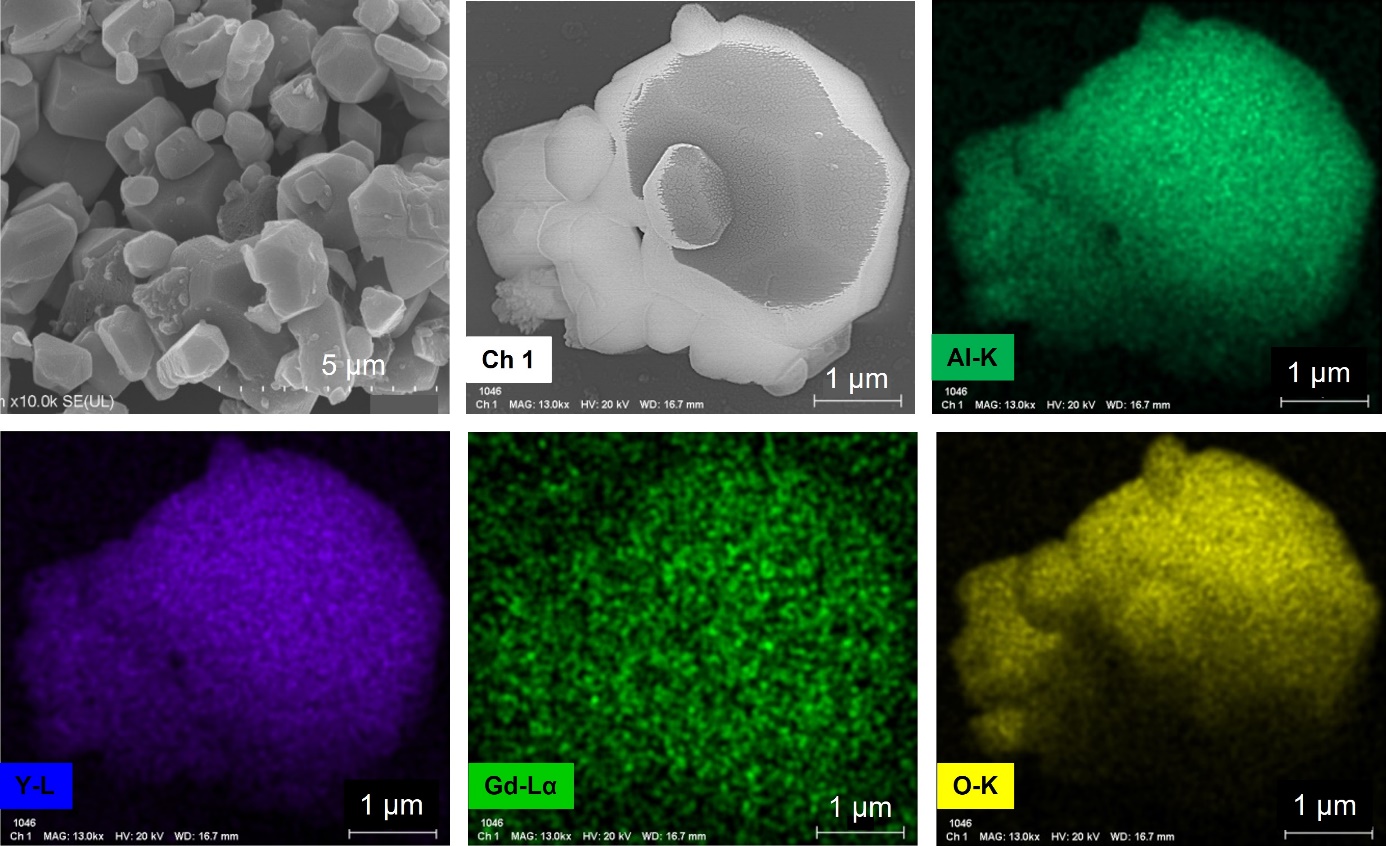


Figure S6. Scanning electron microscope (SEM) images and elemental mapping by energy-dispersive X-ray spectroscopy of YAB: 0.7% Pr^3+^, 20% Gd^3+^.
